# Synergistic role of oral and maxillofacial surgery and prosthodontics in implant-based rehabilitation: a scoping review protocol

**DOI:** 10.1038/s41405-025-00397-7

**Published:** 2026-01-09

**Authors:** Mahesh Mundathaje, Dharnappa Poojary, Jefferson Prince, Sandipan Mukherjee, Naresh Shetty

**Affiliations:** 1 Department of Prosthodontics and Crown & Bridge, Manipal College of Dental Science Mangalore, Manipal Academy of Higher Education, Manipal, India; 2 Department of Oral and Maxillofacial Surgery, Manipal College of Dental Science Mangalore, Manipal Academy of Higher Education, Manipal, India; 3 Clinical Assistant Professor, Oral & Maxillofacial Surgery Department College of Dentistry, Texas A&M University, Dallas, TX USA

**Keywords:** Fixed prosthodontics, Continuing professional development in dentistry

## Abstract

**Objective:**

This protocol for scoping review aims to systematically map existing evidence on collaborative approaches between Oral and Maxillofacial Surgery (OMFS) and Prosthodontics in implant therapy, exploring their implementation, influential clinical domains, current methodologies, technologies, and identifying research gaps.

**Introduction:**

Dental implant therapy has revolutionized patient care, but optimal success, especially in complex cases, hinges on precise surgical and prosthetic foresight. The synergy between OMFS and Prosthodontists is crucial. Modern dentistry embraces a prosthetically driven, integrated paradigm, greatly streamlined by digital workflows. Despite advancements, literature remains fragmented, lacking a comprehensive synthesis of collaborative models. This protocol for review seeks to bridge that gap.

**Inclusion criteria:**

Studies on adult (≥18 years) edentulous or partially edentulous patients undergoing implant-based rehabilitation, focusing on OMFS and Prosthodontics interdisciplinary collaboration in planning, surgery, and prosthetics, will be considered. All clinical settings, regions, and study designs (quantitative, qualitative, mixed methods, reviews, text/opinion papers) are included. Studies solely on a single discipline or maxillofacial prosthetics unrelated to tooth replacement via implants will be excluded.

**Methods:**

This protocol for scoping review explains methodology that will follow PRISMA-ScR guidelines. A comprehensive search will be conducted across PubMed/MEDLINE, Scopus, Web of Science, Embase, and Cochrane Library. Two independent reviewers will screen titles, abstracts, and full texts. Data extraction will use a standardized charting form, followed by descriptive and thematic analysis, presented narratively with tables/figures.

**Review registration:**

Open Science Framework 10.17605/OSF.IO/USZ9V

## Introduction

Dental implant therapy has fundamentally transformed how clinicians approach the management of patients with missing teeth, evolving from rudimentary attempts to highly predictable and widely accepted treatment modalities [1, 2]. Its profound impact lies in its capacity to offer stable, long-term solutions that closely mimic natural dentition, thereby restoring not only essential masticatory function and aesthetics but also significantly improving patients’ oral health-related quality of life [3, 4]. Historically, tooth replacement options were primarily limited to conventional removable prostheses or fixed partial dentures, each with inherent limitations regarding stability, comfort, and the crucial preservation of adjacent healthy tooth structure and the underlying alveolar bone [5]. The groundbreaking advent of osseointegrated implants, a concept pioneered by Brånemark in the mid-20th century, marked a true paradigm shift in dentistry. It provided a biological anchor for prosthetic restorations that directly integrates with the bone, offering unprecedented stability, longevity, and a more natural feel for the patient [6, 7].

However, achieving truly profound and lasting success in the intricate landscape of implant-based rehabilitation, particularly in complex clinical scenarios involving significant anatomical challenges, advanced bone atrophy, or comprehensive full-arch restorations, is rarely the sole accomplishment of a single dental discipline [8, 9]. Instead, optimal and enduring results consistently flourish at the precise intersection of specialized surgical expertise and thoughtful, foresightful prosthetic planning. The collaborative endeavours of Oral and Maxillofacial Surgeons (OMFS) and Prosthodontists are increasingly recognized as absolutely critical for ensuring optimal, stable, and enduring outcomes in contemporary implant dentistry [10, 11]. While the OMFS brings indispensable skills in precise implant placement, advanced bone grafting techniques, and the adept management of surgical complexities, the Prosthodontist contributes invaluable expertise in aesthetic and functional design, meticulous material selection, occlusal harmony, and the crucial long-term maintenance of the prosthesis [12]. This interdisciplinary synergy is far more than a mere sequential handover of treatment steps; it represents an integrated planning and execution process where the desired final prosthetic outcome fundamentally dictates the surgical approach, a concept often termed “prosthetically driven implantology“ [13].

Traditionally, implant treatment often followed a somewhat linear model, where surgical implant placement occurred first, sometimes with insufficient consideration for the nuances of the final prosthetic design. This “surgically driven” approach frequently led to compromises in prosthesis design, aesthetics, and function, often necessitating complex and less predictable prosthetic solutions later in the treatment journey [14]. This historical perspective underscores a significant evolution in implantology, gradually shifting towards a more integrated, “prosthetically driven” paradigm. This modern model ensures that implant positioning is meticulously planned to align optimally with the patient’s specific functional and aesthetic requirements of the final prosthesis, rather than merely adapting the prosthesis to the implants [13, 15]. For instance, Meneghetti et al. demonstrated how detailed digital planning using cone-beam computed tomography (CBCT) and bone reduction guides can streamline complex protocols for two-implant-supported overdentures, emphasizing predictability and interdisciplinary workflow from the outset [16]. Similarly, Sakkas et al. retrospectively evaluated the 5-year outcomes of prosthetically guided implant placement, underscoring how pre-surgical planning led by prosthodontic goals enhances patient outcomes, including implant survival and success rates [17]. Romandini et al, further supported the benefits of minimally invasive, flapless fully guided surgery, indicating improved accuracy and reduced postoperative discomfort, which are direct outcomes of meticulous prosthetically driven planning [18].

The accelerating integration of advanced digital technologies has remarkably streamlined and enhanced this interdisciplinary synergy, significantly improving precision and predictability across all phases of implant therapy, from diagnosis and treatment planning to surgical execution and definitive restoration [19, 20]. Digital dentistry tools have become indispensable in facilitating seamless communication and collaboration between OMFS and Prosthodontists. Intraoral scanners, for example, capture highly accurate 3D models of the patient’s oral cavity, eliminating the need for traditional, often uncomfortable, impressions [21]. This digital data can be immediately shared, facilitating rapid consultation and collaborative planning. CBCT, another cornerstone of digital implantology, provides detailed 3D anatomical information, crucial for assessing bone volume, density, and proximity to vital structures. When combined with sophisticated implant planning software, it allows for virtual implant placement, simulating the ideal position and angulation of implants relative to the planned prosthesis [22, 23].

Furthermore, digital technologies enable the fabrication of highly accurate surgical guides through computer-aided design and manufacturing (CAD/CAM) and 3D printing. These guides translate the virtual plan into precise surgical execution, enhancing the accuracy of implant placement, reducing surgical invasiveness, and improving predictability, especially in complex cases [24, 25]. The ability to virtually design the final restoration and use it as a template for implant placement through “reverse planning” ensures that implants are positioned optimally for prosthetic support, emergence profile, and aesthetics, avoiding complications that can arise from improperly placed implants [26]. Dano et al. presented a case report demonstrating the use of CEREC for prosthetically driven computer-guided implant placement and restoration, highlighting efficiency and predictability [27]. Reddy et al, in a systematic review, explored the effect of digital dentistry on the accuracy of implant placement and prosthesis fabrication, contributing to the understanding of its effectiveness [28]. Banerjee et al. further compared the accuracy in freehand versus computer-assisted implant placement, providing evidence for the superior precision offered by digital guidance [29]. The impact on oral function is also significant; a systematic review and meta-analysis by Srinivasan et al. concluded that the oral function of completely edentulous adults significantly improved with implant-supported/retained prostheses, even when only one jaw received implant therapy, highlighting the importance of this rehabilitation approach [30].

Digital planning technologies have significantly advanced the integration of surgical and prosthetic workflows. The use of CBCT, intraoral scanning, and software platforms that merge DICOM and STL data enables precise virtual planning of implant positions in relation to the definitive prosthesis. This approach improves communication between surgeons and prosthodontists, allowing implant placement to be prosthetically guided rather than solely anatomy-driven. Furthermore, guided surgery and virtual wax-ups enhance predictability, reduce invasiveness by enabling flapless techniques, and shorten treatment times. From a prosthodontic standpoint, digital planning also supports the evaluation of restorative design alternatives, such as cantilevers, which in selected cases may serve as viable substitutes for extensive regenerative procedures. These developments highlight how digital workflows not only strengthen interdisciplinary collaboration but also expand the treatment possibilities for patients with edentulous and partially edentulous arches [31].

Despite these demonstrable advancements and the growing recognition of interdisciplinary collaboration, the current scientific literature on this synergistic relationship, particularly concerning the comprehensive integration of digital workflows, remains somewhat fragmented. While numerous studies address specific surgical techniques or prosthetic innovations, a truly comprehensive synthesis of the collaborative domains, effective clinical models, and the profound impact of integrated digital protocols across both specialties is lacking [19]. This dispersion of knowledge creates a notable gap, hindering a complete understanding of best practices and identifying areas ripe for future investigation. This protocol for scoping review, therefore, endeavours to bridge this gap by systematically mapping the existing evidence on the synergistic relationship between OMFS and Prosthodontics in the nuanced field of implant therapy.

## Review objectives

This protocol for scoping review aims:

1. To map existing evidence on interdisciplinary collaboration between OMFS and Prosthodontics in implant rehabilitation.
2. To identify surgical–prosthetic workflows and communication models reported in the literature.
3. To highlight gaps in evidence and propose future directions.

## Review questions

This scoping review will address the following focused questions:

1. How do Oral and Maxillofacial Surgeons and Prosthodontists collaborate in the planning and execution of implant-based rehabilitation for edentulous and partially edentulous patients?
2. What surgical–prosthetic workflows, communication models, and digital tools have been reported to support interdisciplinary planning and delivery of implant therapy?
3. What knowledge gaps exist in the current literature that require further investigation to optimize interdisciplinary implant rehabilitation?

## Methodology

This scoping review will be conducted in accordance with the Preferred Reporting Items for Systematic Reviews and Meta-Analyses extension for Scoping Reviews (PRISMA-ScR) and the Joanna Briggs Institute (JBI) methodological framework.

## Eligibility Criteria (PCC Framework)

- **Population:** Adults (≥18 years) with edentulous or partially edentulous arches rehabilitated using dental implants.
- **Concept:** Interdisciplinary collaboration between Oral and Maxillofacial Surgery (OMFS) and Prosthodontics during implant planning, placement, and prosthetic rehabilitation.
- **Context:** Clinical, academic, and hospital settings worldwide, without time restriction. Eligible study designs will include clinical studies, case series, reviews, and expert opinions that describe collaborative or prosthetically driven approaches. Studies involving only one specialty or unrelated disciplines will be excluded.

## Information sources

Electronic searches will be performed in PubMed/MEDLINE, Scopus, Embase, Web of Science, Cochrane Library, and Google Scholar. Gray literature (institutional repositories, conference abstracts) and reference lists of included articles will be screened to ensure completeness.

## Search strategy

A comprehensive search strategy using both **MeSH terms** and **keywords** will be developed with librarian support. The strategy will combine terms such as *oral and maxillofacial surgery*, *prosthodontics*, *implant rehabilitation*, *interdisciplinary*, *team-based care*, and *digital planning*. The search syntax will be adapted for each database listed above. The final search strategy for PubMed, including all terms and combinations, is provided in Appendix I.

## Study selection

Two reviewers will independently screen titles and abstracts for relevance. Full texts will be retrieved for potentially eligible studies. Any disagreement will be resolved by discussion or consultation with a third reviewer. The overall selection process will be summarized using a PRISMA-ScR flow diagram.

## Data extraction

A standardized data-charting form will be pilot-tested and used by the same two reviewers. Extracted variables will include author, year, country, study design, sample characteristics, type of implant rehabilitation, description of interdisciplinary workflow, use of digital technologies, reported outcomes, and key conclusions. The final version of the data extraction tool will be provided in Appendix II.

## Data synthesis

Data will be synthesized descriptively. Quantitative results, where available, will be summarized in tabular form; qualitative findings will be organized thematically to illustrate domains of surgical-prosthetic collaboration. No statistical meta-analysis will be undertaken.

## Anticipated limitations

Potential limitations include heterogeneity of study designs, variability in clinical protocols, and possible publication bias. These factors may limit comparability across studies but will not affect the review’s objective to map the scope and nature of existing evidence.

## Ethics and dissemination

This review will use only secondary, published data and therefore does not require ethical approval. Findings will be disseminated through publication in a peer-reviewed journal and conference presentations.

## Conclusion

This scoping review is designed to comprehensively map the existing evidence on how Oral and Maxillofacial Surgery and Prosthodontics collaborate in implant-based rehabilitation of edentulous and partially edentulous patients. By systematically identifying models of interdisciplinary planning, digital workflows, and shared clinical decision-making, the review aims to clarify how a synergistic approach enhances treatment predictability, accuracy, and patient outcomes. The findings are expected to provide an evidence-based foundation for developing integrated clinical pathways, inform curriculum design in implantology training, and highlight key gaps that warrant further empirical research. Ultimately, this review will contribute to strengthening interdisciplinary collaboration in contemporary implant practice and guide clinicians toward more cohesive, prosthetically driven rehabilitation protocols.

## Summary box

### What this review will add

- This scoping review will systematically map current evidence on interdisciplinary collaboration between Oral and Maxillofacial Surgeons and Prosthodontists in implant-based rehabilitation.
- It will identify how digital planning, prosthetically driven workflows, and shared decision-making influence clinical outcomes in edentulous and partially edentulous patients.
- The review will highlight knowledge gaps and propose future research directions to strengthen integrated surgical–prosthetic protocols in implant dentistry.

### Ethical approval

As this protocol for scoping review will synthesize evidence from publicly available, published literature, it does not require ethical approval from an institutional review board. The review will adhere to ethical guidelines for research synthesis, including accurate reporting and appropriate citation of all sources.

## Appendix I Search Strategy

Database: PubMed (via NCBI PubMed)

Date of Search: [July 21, 2025]

**Table:**

Concept	Search Term(s)	Field(s) Searched (e.g., MeSH, Title/Abstract)	Number of Results
1. Oral and Maxillofacial Surgery (OMFS)	“Oral and Maxillofacial Surgery”[MeSH] OR “Oral Surgical Procedures”[MeSH] OR “Oral Surgery”[Title/Abstract] OR “Maxillofacial Surgery”[Title/Abstract] OR “Oral Surgeons”[Title/Abstract] OR “Maxillofacial Surgeons”[Title/Abstract] OR “OMFS”[Title/Abstract] OR “Oral Surgery”[Journal] OR “Journal of Oral and Maxillofacial Surgery”[Journal]	MeSH, Title/Abstract, Journal Name	3979
2. Prosthodontics	“Prosthodontics”[MeSH] OR “Prosthodontic”[Title/Abstract] OR “Prosthodontists”[Title/Abstract] OR “Dental Prosthesis”[MeSH] OR “Dental Prostheses”[Title/Abstract] OR “Removable Partial Dentures”[MeSH] OR “Dentures”[MeSH] OR “Crowns”[MeSH] OR “Bridges”[MeSH] OR “Journal of Prosthetic Dentistry”[Journal] OR “Journal of Prosthodontics”[Journal]	MeSH, Title/Abstract, Journal Name	3171
3. Collaboration / Interdisciplinary	“Interdisciplinary Communication”[MeSH] OR “Interprofessional Relations”[MeSH] OR “Patient Care Team”[MeSH] OR “Collaboration”[Title/Abstract] OR “Collaborative”[Title/Abstract] OR “Interdisciplinary”[Title/Abstract] OR “Multidisciplinary”[Title/Abstract] OR “Teamwork”[Title/Abstract] OR “Team-based care”[Title/Abstract] OR “Integrated care”[Title/Abstract]	MeSH, Title/Abstract	13,385
4. Dental Implants / Rehabilitation	“Dental Implants”[MeSH] OR “Dental Prosthesis, Implant-Supported”[MeSH] OR “Dental Implant”[Title/Abstract] OR “Implant Dentistry”[Title/Abstract] OR “Implant-supported”[Title/Abstract] OR “Osseointegration”[MeSH] OR “Osseointegrated”[Title/Abstract] OR “Tooth Loss”[MeSH] OR “Edentulous Jaw”[MeSH] OR “Edentulism”[Title/Abstract] OR “Oral Rehabilitation”[MeSH] OR “Dental Restoration”[Title/Abstract] OR “Prosthetic Rehabilitation”[Title/Abstract]	MeSH, Title/Abstract	2195
**5. Digital Technologies**	“Computer-Aided Design”[MeSH] OR “Three-Dimensional Printing”[MeSH] OR “Digital Technology”[MeSH] OR “Computers”[MeSH] OR “Virtual Reality”[MeSH] OR “Computer-Assisted”[Title/Abstract] OR “Digital Workflow”[Title/Abstract] OR “CAD/CAM”[Title/Abstract] OR “3D Printing”[Title/Abstract] OR “Virtual Surgical Planning”[Title/Abstract] OR “Intraoral Scan”[Title/Abstract] OR “Cone-Beam Computed Tomography”[Title/Abstract] OR “Guided Surgery”[Title/Abstract]	MeSH, Title/Abstract	4223
Final Combined Search	**(#1 AND #2 AND #3 AND #4 AND #5)**	All Fields	4100
	Date Filter		2006–2025

## Appendix II Draft Data Extraction Tool

Reviewer 1: [Initials] Reviewer 2: [Initials]

Date of Extraction: [DD/MM/YYYY]

**Table:**

Category	Data Item	Notes/Guidance for Extraction	Results
Source Details	Citation (Authors, Year, Journal/Publication Type)	Full reference in Vancouver style. Specify if journal article, book chapter, guideline, dissertation, etc.	
	Country of Origin	Country where the study/source was conducted or authored.	
Study Characteristics	Aim/Purpose of the Source	Briefly describe the main objective(s) of the included source.	
	Study Design / Type of Source	E.g., RCT, Cohort, Qualitative, Mixed Methods, Systematic Review, Clinical Guideline, Narrative Review, Opinion Piece, Textbook Chapter, Conference Proceeding, Dissertation.	
	Patient Population Characteristics	Describe the patient group studied (e.g., completely edentulous, partially edentulous, specific conditions).	
Collaborative Approaches	Models/Protocols of Collaboration	Detail specific ways OMFS and Prosthodontists collaborate (e.g., joint clinics, formalized treatment pathways, shared digital platforms).	
	Communication Strategies/Tools	Describe methods used for communication (e.g., regular meetings, shared patient records, digital communication tools).	
	Team Dynamics / Roles and Responsibilities	How roles are defined, presence of dedicated implant teams, interprofessional education, conflict resolution mechanisms.	
Impact of Collaboration	Reported Treatment Outcomes (Success Rates, Longevity)	e.g., implant survival rates, prosthetic success, bone level changes, esthetic outcomes. Quantify if possible.	
	Patient Satisfaction Levels	Reported measures or qualitative descriptions of patient satisfaction with interdisciplinary approach/outcomes.	
	Incidence of Complications	Surgical, prosthetic, or biological complications related to implant treatment under collaborative care.	
	Efficiency/Streamlining of Care	Reported benefits in terms of treatment time, number of appointments, cost-effectiveness due to collaboration.	
Technological Integration	Specific Digital Technologies Utilized	e.g., Intraoral scanners, CBCT, implant planning software, CAD/CAM, 3D printing, guided surgery, AI in diagnostics/planning, teleradiology.	
	Reported Benefits of Technology in Collaboration	How technology facilitates planning, communication, precision, or outcomes within the interdisciplinary context.	
	Reported Limitations/Challenges of Technology	Barriers to implementation, learning curve, cost, accuracy issues, software compatibility.	
	Levels of Adoption of Technology	Perceived or reported extent of use of specific digital tools within interdisciplinary workflows.	
Challenges and Facilitators	Identified Challenges/Barriers to Collaboration	e.g., communication breakdown, lack of training, ego, logistical issues, differing philosophies, cost.	
	Identified Facilitators/Enablers for Seamless Collaboration	e.g., shared training, mutual respect, standardized protocols, dedicated coordinators, integrated digital platforms.	
Identified Research Gaps	Recommendations for Future Research from Source Authors (related to collaboration/technology synergy)	Note any specific suggestions or questions for future studies on the synergistic role of OMFS and Prosthodontics in implant therapy, particularly concerning digital workflows.	
Additional Notes	(Any other relevant information not captured above)	Space for unique or important findings/observations relevant to the review questions that do not fit into the predefined categories.	

## References

[1] Esposito M, Grusovin MG, Polyzos IP, Felice P, Worthington HV. Interventions for replacing missing teeth: dental implants in fresh extraction sockets (immediate, immediate-delayed and delayed implants). Cochrane Database Syst Rev. 2010;8:CD005968. 10.1002/14651858.CD005968.pub3 PMC12086013 20824846

[2] Tallarico M, Galiffi D, Scrascia R, Gualandri M, Zadrożny Ł, Czajkowska M, et al. Digital workflow for prosthetically driven implants placement and digital cross mounting: a retrospective case series. Prosthesis. 2022;4:654–66.

[3] Papaspyridakos P, Bedrossian A, de Souza AB, Bokhary A, Gonzaga L, Chochlidakis K. Digital workflow in implant treatment planning for terminal dentition patients. J Prosthodont. 2022;31:401–9. 10.1111/jopr.13510 35343618

[4] Conejo J, Sanchez-Lara A, Rivet C, LaMar F. Digital workflow for a definitive implant-supported hybrid prosthesis. Compend Contin Educ Dent. 2024;45:658–62. 39561345

[5] Salomão GVS, Chun EP, Panegaci RS, Santos FT. Analysis of digital workflow in implantology. Case Rep Dent. 2021;2021:6655908. 33628525 10.1155/2021/6655908 PMC7899756

[6] Berzaghi A, Testori T, Scaini R, Bortolini S. Occlusion and biomechanical risk factors in implant-supported full-arch fixed dental prostheses—narrative review. J Pers Med. 2025;15:65. 39997342 10.3390/jpm15020065 PMC11856061

[7] Peitsinis PR, Blouchou A, Chatzopoulos G, Vouros ID. Optimizing implant placement timing and loading protocols for successful functional and esthetic outcomes: a narrative literature review. J Clin Med. 2025;14:1442. 40094901 10.3390/jcm14051442 PMC11900159

[8] Chen CS, Hsu H, Kuo YW, Kuo HY, Wang CW. Digital workflow and guided surgery in implant therapy-literature review and practical tips to optimize precision. Clin Implant Dent Relat Res. 2025;27:292–302. 10.1111/cid.70038 40304451

[9] Kale P, Sen MP, Singh V. Interdisciplinary approaches in modern dentistry: a comprehensive review. J Orofac Health Sci. 2024;16:154–9.

[10] ArkseyH, O’Malley L. Scoping studies: towards a methodological framework. Int J Soc Res Methodol.2005;8:19–32.

[11] Levac D, Colquhoun H, O’Brien KK. Scoping studies: advancing the methodology. Implement Sci. 2010;5:69. 20854677 10.1186/1748-5908-5-69 PMC2954944

[12] Tricco AC, Lillie E, Zarin W, O’Brien KK, Colquhoun H, Levac D, et al. PRISMA extension for scoping reviews (PRISMA-ScR): checklist and explanation. Ann Intern Med. 2018;169:467–73. 30178033 10.7326/M18-0850

[13] Toser I, Faur A, Anghel-Lorinți AE, Jivănescu A. Case report: a comprehensive digital workflow to enhance predictability and precision with fixed dental prostheses in the posterior region. Front Dent Med. 2025;6:1625405. 40630263 10.3389/fdmed.2025.1625405 PMC12234521

[14] Alfayez E. Current trends and innovations in oral and maxillofacial reconstruction. Med Sci Monit. 2025;31:e947152. 40150813 10.12659/MSM.947152 PMC11963826

[15] Ekram AM, Ghazi ME, Abbas SEM, Ekram KM. Full digital workflow for prosthetic driven implant planning and surgical guide fabrication without the need for scan appliance: a case report. J Dent Implant Res. 2024;43:33–8.

[16] Meneghetti PC, Sabri H, Dastouri E, Pereira RMA, Teixeira W, Wang HL, et al. Digital planning for two-implant-supported overdenture and bone reduction guide using cone-beam computed tomography: simple features for predictable outcomes. J Prosthodont. 2023;32:361–70. 36752037 10.1111/jopr.13657

[17] Sakkas A, Westendorf S, Thiele OC, Schramm A, Wilde F, Pietzka S. Prosthetically guided oral implant surgery. A retrospective cohort study evaluating the 5-year surgical outcome. GMS Interdiscip Plast Reconstr Surg DGPW. 2023;12:Doc06. 37693294 10.3205/iprs000176 PMC10486885

[18] Romandini M, Ruales-Carrera E, Sadilina S, Hämmerle CHF, Sanz M. Minimal invasiveness at dental implant placement: a systematic review with meta-analyses on flapless fully guided surgery. Periodontol 2000. 2022;91:89–112. 35906928 10.1111/prd.12440

[19] Elkhadem A, Ahmed MM. Rationale of prosthetically-driven implant placement utilizing implant-navigation system: accuracy validation trial. Egypt Dent J. 2021;67:603–11.

[20] Fernandes G, Aras MA, Coutinho I. Zygomatic implant rehabilitation: a prosthodontic driven approach: a review. Int J Sci Healthc Res. 2024;9:1–5.

[21] Patzelt SBM, Spies BC, Kohal RJ. CAD/CAM-fabricated implant-supported restorations: a systematic review. Clin Oral Implants Res. 2015;26:133–45. 10.1111/clr.12633 26061615

[22] D’Souza KM, Aras MA. Applications of computer-aided design/computer-assisted manufacturing technology in dental implant planning. J Dent Implants. 2012;4:100–4.

[23] Papaspyridakos P, Vazouras K, Chen YW, Kotina E, Natto ZS, Kang KH, et al. Digital vs conventional implant impressions: a systematic review and meta-analysis. J Prosthodont. 2020;29:660–78. 32613641 10.1111/jopr.13211

[24] Kamel AHM, AlKindi F, AlHarrasi R, AlKindi N. The Role of Dental Oncology in Cancer Care: a Critical Component of Comprehensive Treatment, Education, and Interdisciplinary Collaboration- a Narrative Review. J Cancer Educ. 2025; 10.1007/s13187-025-02639-6 [Epub ahead of print]. 10.1007/s13187-025-02639-6 40304875

[25] D’Haese J, Ackhurst J, Wismeijer D, De Bruyn H, Tahmaseb A. Current state of the art of computer-guided implant surgery. Periodontol 2000. 2017;73:121–33. 28000275 10.1111/prd.12175

[26] Flügge T, van der Meer WJ, Gimenez-Gonzalez B, Vach K, Wismeijer D, Wang P. The accuracy of different dental impression techniques for implant-supported dental prostheses: A systematic review and meta-analysis. Clin Oral Implants Res. 2018;29:388–412. 10.1111/clr.13273 30328182

[27] Dano D, Stiteler M, Giordano RA. Prosthetically driven computer-guided implant placement and restoration using CEREC: a case report. Compend Contin Educ Dent. 2018;39:e1–4. 29714496

[28] Reddy NA, Vempalli S, Prakash J, Suganna M, Meenakshi S, Shivakumar GC, et al. Evaluation of the effect of digital dentistry on the accuracy of implant placement and prosthesis fabrication—a systematic review and meta-analysis. Prosthesis. 2023;5:805–24.

[29] Banerjee S, Debnath A, Paul P, Banerjee T. Comparison of accuracy in freehand versus computer-assisted (dynamic and static) dental implant placement: a systematic review and meta-analysis. J Indian Prosthodont Soc. 2025;25:1–10. 40654119 10.4103/jips.jips_369_24 PMC11853947

[30] Srinivasan M, Kamnoedboon P, Angst L, Müller F. Oral function in completely edentulous patients rehabilitated with implant-supported dental prostheses: a systematic review and meta-analysis. Clin Oral Implants Res. 2023;34:196–239. 37750517 10.1111/clr.14068

[31] D’Albis G, D’Albis V, Susca B, Palma M, Al Krenawi N. Implant-supported zirconia fixed partial dentures cantilevered in the lateral-posterior area: a 4-year clinical results. J Dent Res Dent Clin Dent Prospects. 2022;16:258–63. 37560497 10.34172/joddd.2022.041 PMC10407873

